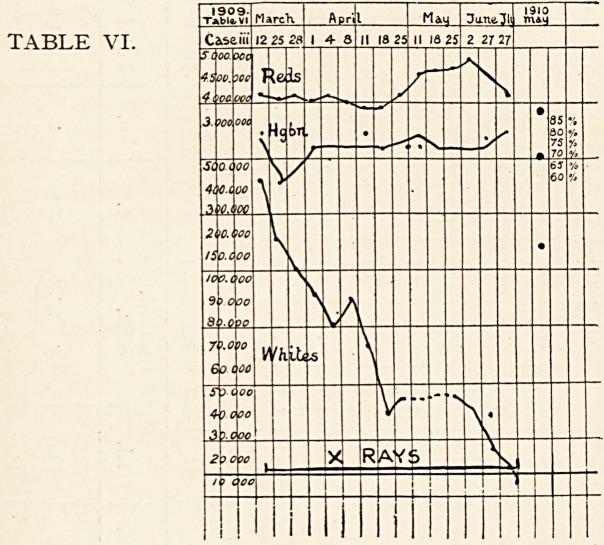# Cases of Leukæmia Treated by X-Rays

**Published:** 1910-09

**Authors:** J. Michell Clarke

**Affiliations:** Joint Professor of Medicine, University of Bristol; Physician to Bristol General Hospital.


### CASES OF LEUKÆMIA TREATED BY X-RAYS.

BY

J. MICHELL CLARKE, M.A., M.D. Cantab., F.R.C.P.,

*Joint Professor of Medicine, University of Bristol;*
*Physician to Bristol General Hospital.*

---

THE beneficial effects of the X-rays in the treatment of leukæmia are now well known; unfortunately, they do not seem to be permanent. Although a cure is not to be expected, very decided improvement results for a considerable time. Cases of myeloid leukæmia do best. The most striking effect of the X-rays is a rapid reduction of the number of white cells, especially of myelocytes, in the blood, generally, but not invariably, accompanied by a remarkable shrinking of the enlarged spleen and a concomitant improvement in the general condition. The patients soon feel stronger and lose the sense of lassitude which so often attends this disease, or they feel more easy from the reduction of the splenic tumour. The subjective sense of improvement often precedes the other evidences of benefit from the treatment.

As to the mode of action of X-rays in leukæmia, the interpretation will necessarily vary according to the view held of the pathology of the disease.

It is now generally accepted that the marrow is affected in all cases of leukæmia, and is the chief seat of the enormous production of white cells. There is less unanimity as to the part played by the spleen in myeloid, and by the lymph-glands in lymphatic leukæmia. It is known that the spleen sorts out from the blood and destroys effete leucocytes. Dr. Andrewes in his recent Croonian Lectures states that "the opinion that the spleen-pulp is the grave of the leucocytes is fully confirmed from my observations; in no other tissue have I found these disintegrating leucocytes." The enlargement of the spleen in leukæmia may be primarily due to its attempting to carry out its normal functions and to sort out and destroy the ever-increasing number of white cells in the blood. The pathological changes in the splenic structures may follow these efforts, partly from the task being beyond its powers and partly from the toxic products of disintegrating cells.

It is conceivable that the X-rays may act either by preventing the excessive formation of leucocytes and myelocytes, or by destroying them after they are formed (leucolysis), or by preventing them from getting into the blood. The last hypothesis may probably be dismissed at once, though from the known destructive action of the X-rays upon enzymes it has been suggested that their action in leukæmia depends upon the destruction of an enzyme in the blood, which exerts a chemiotactic action upon the cells in the marrow.

With regard to the hypothesis that they prevent the excessive formation of white cells, Mosso and Milchener found that the action of X-rays on the marrow of rabbits was to cause a partial destruction of the white cells of the marrow, whilst the reds, nucleated and non-nucleated, were not affected. This, however, could be claimed as a leucolytic action. According to Harris[1] a leucolytic action of the rays is the explanation which on the grounds of experiment has most in its favour, although it is not known whether the leucolytic body or bodies prevents an excessive formation of leucocytes or destroys them after they are formed. He quotes experiments of Helber and Linser, who produced leucopenia by injection of serum from an animal exposed to the X-rays, and observations by Capps and Smith,[2] who found that serum from leukæmic patients, who were improving under X-ray treatment, caused leucopenia when injected into animals, and again, when injected into another case of leukæmia not treated by X-rays, produced a rapid and decided drop in the number of leucocytes in the latter.

Taking the view given above as to the rôle of the spleen in leukæmia, we may conclude that the effect of the X-rays is by the development of leucolytic bodies to destroy the cells accumulated in the spleen. It may be remarked in passing that

[1] H. Harris, "Myelogenous Leukemia and its Treatment with X-rays," *Am. J. M. Sc.*, 1908, cxxxvi, 78.

[2] *J. Exper. Med.*, 1907, ix, 51.

another point in favour of a leucolytic action of X-rays is the presence of degenerated white cells in the blood, as will be seen in the cases given below. Further, this action on the spleen would explain the rapid fall in the number of white cells, the diminution in the size of the organ, and also another fact shown in Cases 2 and 3, namely that when after a time the effect of the X-rays was lost the blood deteriorated first, and the characteristic leukæmic blood-picture returned before the spleen again became much enlarged. To my mind this points to the splenic tumour being a secondary phenomenon in leukæmia, but as patients do not come for advice in the first place before the spleen is large, it

TABLE I.

is a point which we are not in a position to determine under ordinary circumstances.

I will now give an account of four cases of leukæmia treated by X-rays.

**Case 1.**—A.B., æt. 24, car-driver, admitted to hospital October 13th, 1905. The family history was good, and the patient himself had never had any bad illness except pneumonia twenty years previously. Twelve months before admission he had for a few days a dull, aching pain in the left hypochondrium; he then remained well until five months later, when he noticed swelling of the abdomen and a hard lump just below the left ribs. The lump steadily increased in size, and six weeks before admission he sought medical advice. The swelling was not painful. During the last three months he had lost flesh, had vomited occasionally, had a few attacks of epistaxis, and a hard, dry cough.

Aspect, healthy; temperature, normal; appetite, good; bowels, regular; tongue, clean; lungs, normal; heart, apex beat in normal position; area of dulness, normal; sounds, irregular, with a faint systolic murmur at apex and no thrill; pulse, 54, irregular.

Abdomen, large; edge of liver, smooth, and felt one inch below ribs; spleen, very large, firm, smooth and hard. Its vertical diameter (below ribs) was $7\frac{1}{2}$ inches, and transverse (through umbilicus) 8 inches. No ascites, no œdema of legs.

DIFFERENTIAL COUNT OF WHITE CELLS (PERCENTAGES).
TABLE II. CASE 1.

| Date. | pmn. | s.l. l.l. | t. | e. | b. | f.g. e. my. my. | Undiffd. cells and l.m. | Total Whites. | Total myelocytes. |
|---|---|---|---|---|---|---|---|---|---|
| Oct. 15 | 46 | 10 8 | | 6 | | 20 | 10 | 62,000 | 20% |
| 30 | 44 | 13 | | 3 | | 28 | 12 | 74,000 | 28%† |
| Nov. 7 | 48 | 11 | | 4 | | 27 | 10 | 110,000 | 27%† |
| 27 | 57 | 8 | | 5 | | 23 5 | 2 | 50,000 | 28%† |
| Dec. 8 | 63 | 15 | | 2 | | 17 | 3 | 16,000 | 17%† |
| 20 | 71 | 18 2 | | 3 | | 1 | 5 | 12,000 | 1%† |
| Jan. 1 | 65 | 21 | 1 | 2 | 4 | 3 | 4 | 39,000 | 3% |
| 18 | 62 | 20 | 2 | | 4 | 7 | 4 | 64,000 | 7% |
| 25 | 62 | 15 | | 2 | | 11 4 | 4 | 66,000 | 15% |
| Feb. 5 | 50 | 16 | | 2 | | 26 | 6 | 87,000 | 26% |
| 26 | 51 | 15 | | 4 | 1.3 | 12.7 | 7 | 108,000 | 22.7% |

† X-ray treatment.

To save space, the following abbreviations are used in the tables of differential counts :—

pmn. = polymorphonuclears.
s.l. = small lymphocytes.
l.l. = large lymphocytes.
t. = transitionals.
e. = eosinophils.
b. = basophil, or mast cells.
f.g. my. = myelocytes with fine granules (amphophilic).
deg. my. = degenerate myelocytes.
e. my. = eosinophil myelocytes.
b. my. = basophil myelocytes.
l.m. = large mononuclears.
nblts. = normoblasts.
mblts. = megaloblasts.

Urine, sp. gr. 1020 acid, contained a trace of albumin and no sugar; it deposited no uric acid. For condition of blood see Tables 1 and 2.

In this case the X-rays were applied over the spleen only three times a week. He soon began to feel much better, and was able to get up and move about. There was a fall in the red cells for about three weeks, which then returned to normal and remained so.

During the first four weeks of treatment the white cells steadily increased in number, reaching the highest point five weeks after it was begun; there was then a somewhat rapid fall, which lasted for a month and continued for a month after the X-rays were stopped. The fall was from a total count of white cells of 90,000 per c.m.m. to 12,000. At the end of this period the number began to rise, and continued to do so until he left the hospital in February 26th, 1906, when they reached a total of 108,000 per c.m.m. This was the first case treated, at a time when there had been less experience of the action of X-rays than is now the case, and the applications produced much redness and soreness of the skin, so that they had to be discontinued. The spleen was very slightly, if at all, diminished in size under their use, and the effect on the white cells, though profound for a short time, was very temporary. There was an improvement in general health. I heard of his death about eighteen months after he left the hospital. A copious deposit of urates, but no uric acid crystals, was observed in the urine during treatment. There was no increase in the trace of albumin found on admission, and no casts were seen.

**Case 2.**—July 18th, 1908. S.B., a hard-working woman, aged 42, has had three children, comes of a healthy family, and had always enjoyed good health until two years before, when she suffered for a short time from swelling of the hands and knees. She sought admission to the hospital for dyspeptic pains, vomiting, and aching pains in the abdomen, which were relieved by rest. Catamenia ceased suddenly two years before.

Aspect, healthy, well nourished; pulse, 84; respirations, 20; temperature, 99°; appetite, bad; bowels, regular; tongue, moist and clean. The urine, sp. gr. 1022; daily quantity about 40 oz.; urea deficient in amount, 1 per cent. It contained no albumin or sugar, and deposited no uric acid crystals. Except for a soft systolic murmur at the apex, the heart and lungs were normal.

Abdomen, large; liver, edge smooth, about 1 inch below costal margin; spleen occupied nearly the whole of the left side of the abdomen, extending to within 2 inches of the umbilicus, and 1 inch above the iliac crest. It was smooth, firm, not tender. Splenic dulness began above at seventh rib. No ascites.

The bones and joints appeared normal, except for some tenderness over the right tibia.

From July 21st to August 16th she was treated with injections of atoxyl, gr. ij daily. As there was no improvement and she had an attack of diarrhœa this was stopped.

On September 8th treatment by X-rays was begun. In a week's time there was a marked improvement in strength and general well-being. For duration of X-ray treatment and effect on blood condition see Tables 3, 4 and 5. During the first three weeks of treatment the urine became loaded with urates, and there was a marked increase in uric acid passed. On October 7th she left the hospital, and was treated as an out-patient with

TABLE III.

X-rays until the end of December. With the improvement in the blood the spleen diminished in size, so that finally its tip only was felt about 2 inches below the ribs.

From January to April, 1910, she took a course of arsenic. From November, 1908, to September, 1909, she remained in good health, and was able to resume an active life, do all her housework, and also earn her living as a charwoman. In September, 1909, her health began again to deteriorate, with the reappearance of leukæmic changes in the blood, but without any further enlargement of the spleen. A second course of X-rays was given from November 4th, 1909, to March 10th, 1910, but as will be seen from the chart, without influence on the blood-state, which

TABLE IV. CASE 2.—DIFFERENTIAL COUNT OF WHITE CELLS (PERCENTAGES).

| Date. | pmn. | s.l. | l.l. | t. | e. | b. | f.g. my. | deg. my. | e. my. | b. my. | Undiffd. lymphoid cells and mono-nuclears. | nblts. mblts. 500 Whites. | my. % | Total Whites. | | |
|---|---|---|---|---|---|---|---|---|---|---|---|---|---|---|---|---|
| 1908. July 19 | 33 | 4 | 2.2 | 2.2 | 2.2 | 2.2 | 34.9 | | 4 | ·7 | 6 | 4.8% | 39.6 | 324,700 | Atoxyl. | |
| 26 | 40.2 | 3.4 | 1.6 | ·3 | 2.2 | 1.8 | 36 | | 6.8 | 2.3 | 5 | ·9% | 45 | 270,000 | | |
| Aug. 31 | 26.6 | 2.8 | 2.6 | 4 | 3.8 | 1.2 | 47.8 | | 5.6 | 4.4 | 2 | 2.2% | 57.8 | 285,000 | | |
| Sept. 4 | 33 | 2.4 | 1.8 | 1 | 1 | ·5 | 38.6 | | 5.7 | 1.7 | 11 | 11 | 45.9 | 333,000 | X-rays. | |
| 28 | 31 | 2.6 | ·4 | 4.2 | 2.4 | 2 | 47 | | 6 | 2 | 3 | 3% | 55 | | | Oct. 21 to Nov. 4, X-rays stopped. Tip of spleen only to be felt in descent. |
| Oct. | 40.8 | 3.6 | 2.4 | 10 | 3 | ·2 | 31.6 | | 2.2 | 2.8 | 1 | 2 | 38.6 | 127,000 | X-rays 48 times. | |
| Nov. | 45.3 | 4.8 | 1 | 6.2 | 2.4 | 2.2 | 27.7 | | 13. | 1 | 4.8 | 1.5 | 30 | 60,000 | | |
| Dec. | 47.8 | 15.3 | 7.4 | 3.4 | 3 | 3 | 13 | | 2.6 | 1.4 | 4 | 1 | 17 | | | |
| 1909. Jan. | 60 | 16 | 4.5 | 2 | 7.5 | 5 | 1.5 | | 2 | ·5 | 1.5 | ·2 | 4 | 17,000 7,200 | Spleen remained small. | |
| Feb. | 65 | 18.6 | 8.6 | ·4 | 3.8 | 2.2 | ·8 | | | | 2 | | ·8 | 12,000 | | |
| Mar. | 60 | 18 | 7.5 | ·2 | 5.8 | 3 | ·9 | | | | 2.5 | ·3 | ·9 | 12,400 | | |

TABLE IV. CASE 2.—DIFFERENTIAL COUNT OF WHITE CELLS (PERCENTAGES).

| Date. | pmn. | s.l. | l.l. | t. | e. | b. | f.g. my. | deg. my. | e. my. | b. my. | Undiffd. lymphoid cells and mono-nuclears. | nblts. | mblts. 500 Whites. | my. % | Total Whites. | | |
|---|---|---|---|---|---|---|---|---|---|---|---|---|---|---|---|---|---|
| 1909. June | 64.6 | 19 | 5 | 2.5 | 2.6 | 2.7 | 1 | | | .2 | 2 | | | 1.2 | 12,400 | | |
| July | 56.5 | 18.7 | 2.6 | 4 | 2.6 | 2 | 5.4 | | 1.4 | 1.3 | 5.2 | | | 7.9 | 12,000 | | |
| Sept. | 50.2 | 15.4 | 1.2 | 3.5 | 5.5 | 5 | 10.7 | | .2 | .7 | 5.2 | | | 11.6 | 31,800 | | Spleen remained small. (June 1909–Jan. 1910) |
| Oct. | 52.5 | 7.5 | 4.4 | 3.8 | 2.2 | 1.5 | 15.4 | | .7 | .1 | 10 | | | 16.2 | 28,800 | | |
| Dec. | 33.5 | 12.5 | 4.2 | 2.7 | .75 | 5 | 16.5 | 12.2 | 1.7 | 1.7 | 7.5 | | | 32 | 69,600 | | |
| 1910. Jan. | 36 | 6.3 | 1 | 3.6 | 1 | 2.3 | 16 | 27.3 | 1 | 1 | 4.3 | .3 | | 45 | 73,000 | X-rays 40 times. (Oct. 1909–Mar. 1910) | |
| Feb. | 40 | 3.1 | .6 | 4 | 1.5 | 3.4 | 10.3 | 18.7 | 4.7 | 2.2 | 11.5 | ·3 | | 35.9 | | | |
| Mar. 1 | 35.5 | 5 | 3.6 | 3 | 1.6 | 4 | 11.3 | 18.2 | 2.8 | 2.5 | 11.4 | ·3 | | 34.9 | 129,600 | | |
| May 1 | 38.5 | 3 | 2.4 | 5 | 1.4 | 2.4 | 32.4 | | 3.6 | 1.6 | 7.6 | 3.8 | | | 232,000 | | Spleen very slightly in-creased. (Feb.–June 1910) |
| June 1 | 34.6 | 3 | .44 | 4.6 | 1.3 | 3.7 | 16.4 | 27 | 2.2 | 2 | 4.6 | 4.6 | | 47.6 | 146,000 | | |

became worse, but never so bad as on first admission. As she felt weak and ill, she was admitted to hospital in February. There being no signs of improvement under the X-rays, these were stopped on March 10th. The spleen began to slowly enlarge again at the end of February, and is now about 4 to 5 inches below the ribs, about a third of its size on first admission. In general condition and strength she improved with rest in bed and a course of arsenic in moderate doses, and was able to leave the hospital in May. She is now in moderate health, is fat, suffers no pain, but is weak, complains of shortness of breath, and is not able to do very much.

TABLE V. CASE 2.

TOTAL NUMBER OF DIFFERENT FORMS OF WHITE CELLS PER C.M.

| | Total Whites. | January, 1909. | October, 1909. | May, 1910. |
|---|---|---|---|---|
| July, 1908 .. .. | 270,000 | 9,200 | 28,800 | 232,000 |
| pmn. .. .. .. | 108,000 | 6,080 | 15,120 | 86,220 |
| s.l. .. .. .. | 9,180 | 1,656 | 2,160 | 6,960 |
| l.l. .. .. .. | 4,000 | 791 | 1,267 | 5,568 |
| e. .. .. .. | 5,940 | 349 | 638 | 3,248 |
| b. .. .. .. | 4,200 | 213 | 432 | 5,568 |
| f.g. my. .. .. | 97,200 | 92 | 4,455 | 74,168 |
| e.m. .. .. .. | 15,120 | | 201 | 8,252 |
| b.m. .. .. .. | 5,950 | 8.5 | 28.8 | 3,400 |
| Undiffd. .. .. | 13,500 | 184 | 2,880 | 1,753 |
| Transitional .. | 900 | 340 | 1,100 | 11,600 |

**Case 3.**—Seen March 12th, 1910. J. B., a man, aged 53. He had always enjoyed good health, and had never had syphilis or any severe illness. Four months previously he lost the left eye from irido-cyclitis, and since then had not taken his usual amount of exercise. Lately he had felt weak with a feeling of weight in the left side of abdomen, and whilst being examined during an attack of vomiting the large spleen was discovered. He was a stout and healthy-looking man.

Appetite, good; bowels, regular; tongue, clean; pulse, 80, regular; temperature, normal; thoracic organs, normal. No enlarged glands anywhere detected. Abdomen was prominent and full. The liver, enlarged, smooth, not tender, its edge came 3 inches below the ribs. Spleen very large, smooth, firm, not painful, it occupied nearly the whole of the left side of abdomen, reaching to iliac crest below, and to 1 inch from umbilicus; it measured $7\frac{1}{2}$ inches in vertical (nipple) line from lower border of ribs and $10\frac{3}{4}$ inches in transverse diameter. No ascites. The condition of the blood is given in the charts.

On March 19th treatment by X-rays was begun. The case was complicated by glycosuria. The urine, 30 ounces per diem,

TABLE VI.

sp. gr. 1035, no albumin; sugar, 9 per cent.; urea, 2 per cent. After X-rays copious deposit of urates and some uric acid crystals. No diacetic acid or acetone present.

On April 1st he felt a difference in the size and weight of the spleen. Its anterior edge was now 3 inches from umbilicus, vertical diameter $5\frac{1}{2}$ inches from lower border of ribs, transverse $9\frac{1}{2}$ inches. Urine, 40 ounces, sp. gr. 1030, copious deposit of urates, no uric acid crystals. Urea, 3.5 per cent., no albumen, no sugar. The disappearance of sugar was due to his having been placed on a strict diet. No acetone or diacetic acid. Treatment by X-rays was continued until May 19th. The patient's general condition steadily improved during this time. The sp. gr. of the urine fell to about 1020; it contained no albumin or

TABLE VII. CASE 3.—DIFFERENTIAL COUNT OF WHITE CELLS (PERCENTAGES).

| Date. | pmn. | s.l. | l.l. | t. | e. | b. | f.g. my. | deg. my. | e. my. | b. my. | Undiffd. lymphoid and l.m. cells. | Nuclear red cells. | my. % | Total Whites. | | |
|---|---|---|---|---|---|---|---|---|---|---|---|---|---|---|---|---|
| 1909. Mar. 12 | 30 | 3.5 | 3 | 2.8 | 2.7 | 4.5 | 47.5 | | 2.6 | | 5 | 3.5% | 49.5 | 429,160 | Spleen occupied left half abdomen, gradually diminished until tip only felt. | |
| 25 | 43 | .7 | .4 | 3 | 1.4 | 2 | 40.7 | | 2.8 | 1.6 | 3.3 | 2.5% | 45 | 191,875 | | X-rays twice a week. |
| April 1 | 48.2 | 2.4 | 1.2 | 4 | 1.7 | 4 | 32 | | 1.2 | 1 | 4.2 | 0 | 34.2 | 96,875 | | |
| 4 | 57.6 | 2.8 | 1.6 | 5.4 | 2.6 | 6 | 20.5 | 4 | .4 | 0 | 5.2 | 1 | 24.9 | 80,000 | | |
| 8 | 47.8 | 2.8 | 1.2 | 3.2 | 1.6 | 6.8 | 32.8 | | 1.4 | .2 | 4.2 | | 34.1 | 93,750 | | |
| 11 | 47.2 | 3.2 | 1.2 | 4.2 | 1.4 | 5.8 | 24.4 | 4.8 | 1.4 | .6 | 6 | 1% | 31 | 73,500 | | |
| 18 | 50 | 5 | 1.2 | 4.5 | 1.8 | 5 | 21.8 | 4.5 | .3 | .2 | 5 | | 26.8 | 42,000 | | |
| 25 | 54.4 | 5.2 | 1.4 | 3.6 | 1.6 | 6.4 | 18 | 4.6 | .9 | .2 | 3.6 | 1.5% | 23.7 | 49,000 | | |
| May 2 | 64.6 | 3.4 | .9 | 1.1 | 1.6 | 9 | 13 | 1.3 | .2 | .2 | 3.2 | .4 | 14.7 | 39,000 | May to July X-rays once a week. | |
| July 27 | 75.6 | 14.6 | 4.6 | 2 | 2.2 | .4 | 2. | | | | .6 | 0 | .2 | 13,000 | | |
| 1910. May 13 | 43 | 2 | 2 | 2.8 | 2.5 | 2 | 31 | 5.2 | 3 | 1.4 | 5 | .5 | 40 | 131,440 | Spleen as at first. | |

| | Date. | pmn. | s.l. | Undiffd. cells. | t. | e. | Total Whites. |
|---|---|---|---|---|---|---|---|
| CASE 4. | Nov. 15.. | 26.6 | 32.6 | 18.7 17.7 (37.4) | 2.5 | 1 | 14,000 |
| | April 19.. | 23.5 | 36 | 33.75 | 4.25 | 1.5 | 7,500 |

sugar, and the urates and uric acid, at first deposited in large quantities, disappeared. The quantity passed averaged 50 ounces daily, and contained from 3 to 4 per cent. urea. The spleen on May 2nd measured 5 inches in the vertical (from lower border of ribs) and 8 inches in transverse diameter. There was considerable bronzing of the skin over the body generally, especially over the abdomen and splenic regions, over the area where X-rays were applied. On July 27th he was in very fair health, and the tip of the spleen only could be felt about 2 inches below the ribs. He remained well for some time, and was not seen again until May 13th, 1910. He had then been gradually going back for the previous two or three months, feeling drowsy and languid. Tables 6 and 7 show the deterioration of the blood-state. There was now slight œdema of the legs. The knee-jerks were absent. He had relaxed the dietary restrictions, and the urine contained 10 per cent. sugar, a faint trace of albumin; no acetone or diacetic acid. The spleen had again greatly enlarged, its anterior edge being 2½ inches from umbilicus, lower edge 1 inch above iliac crest, measuring 6½ inches in vertical and 10½ in transverse diameter. No ascites. Liver as before. He now resumed treatment once a week by X-rays.

For comparison with the foregoing cases of myeloid leukæmia, the following case of a not very common form of chronic lymphatic leukæmia (lymphæmia) is interesting.

**Case 4.**—E. B., aged 46, an athletic man leading a healthy country life, was seen in November, 1909. Some years ago he had an attack of gout, and since then has kept to a very restricted diet, perhaps insufficient for his needs. He occasionally gets sore throat, but has been better in that respect lately. Five months previously he first noticed enlarged glands on the left side of his neck. No other illness. Appearance, healthy; thoracic and abdominal organs, healthy; spleen not felt; no ascites; urine normal. Numerous enlarged glands were felt on both sides of neck, superior and inferior cervical glands, mostly about size of hazel-nut to almond; some were as small as a pea, firm, hard, discrete, not tender. Skin over enlarged glands normal. Similar enlarged glands were found in both axillæ, groins, and on both sides of the chest. There was no evidence of enlargement of mediastinal or abdominal glands. No tenderness of long bones.

*Blood.*—Red cells, 3,800,000 to c. mm.; hæmoglobin, 75 per cent. The number of whites was not large, 10,000 to c.m.m. Table VII, Case 4, gives the differential count, which was characteristic of lymphatic leukæmia. The mononuclears comprised 70 per cent., of which nearly half had the characteristics of the small lymphocyte; the others were larger, corresponding to the undifferentiated lymphoid cell, or characteristic cell of lymphatic leukæmia. In about half of these the nucleus was mulberry-shaped, indented or nodular, and in many was actively dividing. The very large mononuclears found in acute lymphæmia were absent.

*April*, 1910.—He had been treated by X-rays to the glands once a week for three months. On account of his work keeping him in the country, he had not been able to get the treatment more often. In addition, he had taken arsenic for one month, but had then been obliged to desist from digestive disturbance, and then took phosphorus. He had kept fairly well, and gained one stone in weight. He had had an occasional slight epistaxis. The enlarged glands were still to be felt in the same situations, but were decidedly smaller and harder. Examination of blood gave red cells 3,100,000, whites 8,000 to c.m.m.. Hæmoglobin, 80 per cent. The differential count of whites is given in the table. It is practically unchanged, the mononuclears being 69.75 per cent. The effect of the X-rays in this case has been that the total number of whites has diminished, possibly due to destruction of cells in the glands; but their essential character, and departure from the normal, remained unaltered. The fall in the number of red cells, though not very great, was sufficiently marked to be an unfavourable omen. The X-rays had been discontinued for about eight weeks previous to my second examination, and therefore their effect should have passed off if the diminution in the reds was due to them.

With regard to the mode of application of the X-rays, Dr. W. K. Wills, to whom I am much indebted for his careful treatment of the first three cases, tells me that Case 2, from September 12th to December 8th, 1908, was treated by forty-eight exposures with the high tube at 6-in. distance, for ten minutes at a time daily over the spleen and long bones of extremities. Screens were used, and the rays filtered through six layers of thick boiler felt. The second course was forty-one exposures, on alternate days, of a third of a pastille dose over spleen, arms, legs and spine.

Case 3 was treated from March 20th to May 19th twice each week, for ten to twelve minutes each time, over the splenic area and long bones, through the ordinary clothes, with the high tube (alternating spark gap six inches).

In both cases the first improvement was in loss of the feeling of lassitude and fatigue on slight exertion. There was a gain in strength and general well-being. The effect on the blood condition can be seen from the charts, which show a rapid diminution in all kinds of white cells. With the fall in the number of myelocytes the polymorphonuclears, small and large lymphocytes, and in less degree the eosinophils, show a relative increase, and a more or less complete return to their proper proportions in the differential count. This can also be seen from Table V, which gives the total numbers of white cells respectively per c.m.

With regard to the spleen, in both cases it offered a remarkable diminution in size, and this occurred with the fall in the number of white cells. This is not a constant feature in the treatment of leukæmia by X-rays. As the first case shows, there may be little or no alteration in the splenic tumour. Further, as the condition of the blood began again to revert to the leukæmic state, the spleen slowly began to enlarge, but in Case 2 the blood-condition showed a marked deterioration before the spleen gave decided evidence of fresh enlargement. It is perhaps legitimate to infer from this that the morbid blood-state advances to some degree in myeloid leukæmia before there is any marked enlargement of the spleen. A further point to be noticed is that the resumption of treatment by X-rays in Case 3 after the relapse had practically no effect.

Case 3 was complicated with glycosuria of considerable amount, probably an accidental complication. I have not met with it before in leukæmia. The glycosuria readily cleared up on cutting off carbohydrates from the diet. It seemed to make no difference to the X-ray treatment. On account of the large proportion of proteid in the diet, the urine whilst he was under the X-rays deposited an enormous amount of amorphous urates. The urea varied from 3 to 4 per cent., and the quantity of urine averaged from 45 to 50 ounces daily. No acetone or diacetic acid was present.

Estimated on the seventh and fourteenth days after the commencement of X-ray treatment, the amounts of N. in the food were respectively 17.6 and 22.3 grammes, and in the urine estimated as urea, 13.5 and 20 grammes. On the second occasion he was on a full diabetic diet, the total calories being 2,397, and his weight 67 kilos. The weight remained stationary at this level whilst under treatment.

To the above remarks about the blood it should be added that during the treatment by X-rays a very large number of degenerate white cells appeared in the blood, both myelocytes and polymorphonuclears. Other cells of both these varieties appeared to be irregularly and imperfectly formed. From both causes it was sometimes difficult to know to which group to assign a particular cell. The altered polymorphonuclears were comparatively to the myelocytes easy to recognise. Some of the polymorphonuclears showed fragmentation of nucleus, irregularity in size and shape of cells, and variations as regards granules, Many of the myelocytes showed irregularity in size and shape of nuclei, many being deeply indented, multilobular, or distorted. The cell bodies were irregular, a few contained azure granules, others were vacuolated, and some were faintly-staining phantoms, or had a film-like, disintegrating cytoplasm. The red cells showed less change, but there were many small cells, and some variation in depth of staining. Some of the eosinophil cells showed a few large granules staining deep purple amongst the ordinary granules.

In conclusion, these cases confirm the experience of other observers, that although X-ray treatment does not, unfortunately, result in cure in leukæmia, it gives a greater measure of relief in most cases than is derived from any other form of treatment, and prolongs life. If ordinary precautions are taken, and a careful watch kept on the state of the blood as regards the red cells and hæmoglobin, and on the condition of the urine throughout the period of treatment, it seems to be devoid of danger. The presence of a slight albuminuria in the first case and of marked glycosuria in the third seemed to form no contra-indication to X-ray treatment, as they gave no trouble. The experiments of Mosso and Milchener quoted above show that the X-rays should be applied over the long bones and vertebræ as well as over the spleen.

I am much indebted to Professor Emrys-Roberts for his aid in the blood examinations.

## Figures and Tables

**TABLE I. f1:**
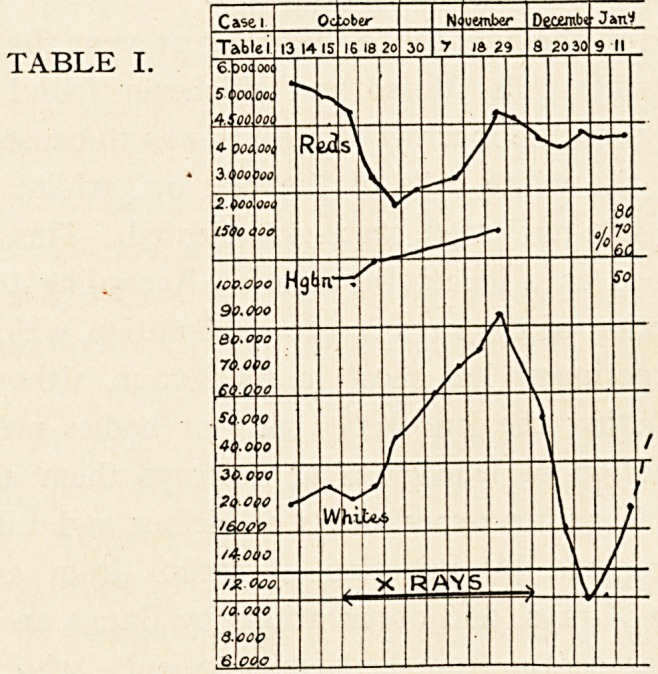


**TABLE III. f2:**
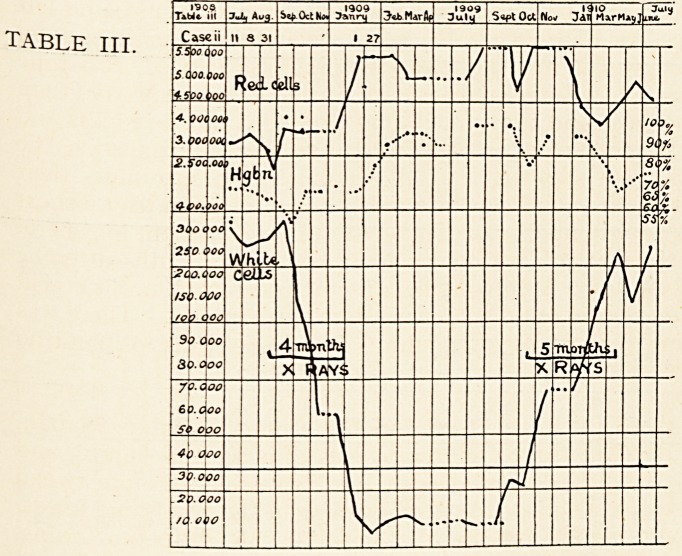


**TABLE VI. f3:**